# Radium-223 dichloride bone-targeted alpha particle therapy for hormone-refractory breast cancer metastatic to bone

**DOI:** 10.1186/2162-3619-3-23

**Published:** 2014-09-08

**Authors:** Amol Takalkar, Scott Adams, Vivek Subbiah

**Affiliations:** 1 PET Imaging Center, Biomedical Research Foundation of Northwest Louisiana, Shreveport, LA 71103, USA; 2 Department of Investigational Cancer Therapeutics (Phase I Clinical Trials Program), Division of Cancer Medicine, The University of Texas MD Anderson Cancer Center, Houston, TX 77030, USA

**Keywords:** Radium 223, Xofigo, Breast cancer, Bone metastases, Alpha particle, Unusual responder, Outlier responder, Exceptional responder

## Abstract

**Background:**

Hormone-refractory breast cancer metastatic to bone is a clinically challenging disease associated with high morbidity, poor prognosis, and impaired quality of life owing to pain and skeletal-related events. In a preclinical study using a mouse model of breast cancer and bone metastases, Ra-223 dichloride was incorporated into bone matrix and inhibited proliferation of breast cancer cells and differentiation of osteoblasts and osteoclasts (all *P* values < .001) *in vitro*. Ra-223 dichloride also induced double-strand DNA breaks in cancer cells *in vivo.*

**Methods:**

The US Food and Drug Administration recently approved radium-223 (Ra-223) dichloride (Ra-223; Xofigo injection) alpha-particle therapy for the treatment of symptomatic bone metastases in patients with castration-resistant prostate cancer. On the basis of a strong preclinical rationale, we used Ra-223 dichloride to treat bone metastases in a patient with breast cancer.

**Results:**

A 44-year-old white woman with metastatic breast cancer who was estrogen receptor–positive, *BRCA1*-negative, *BRCA2*-negative, *PIK3CA* mutation (p.His1047Arg) positive presented with diffuse bony metastases and bone pain. She had hormone refractory and chemotherapy refractory breast cancer. After Ra-223 therapy initiation her bone pain improved, with corresponding decrease in tumor markers and mixed response in ^18^F-FDG PET/CT and ^18^F-NaF bone PET/CT. The patient derived clinical benefit from therapy.

**Conclusion:**

We have shown that Ra-223 dichloride can be safely administered in a patient with hormone-refractory bone metastasis from breast cancer at the US FDA–approved dose for prostate cancer. Furthermore, because the treatment did not cause any drop in hematologic parameters, it has the potential to be combined with other radiosensitizing therapies, which may include chemotherapy or targeted therapies. Given that Ra-223 dichloride is already commercially available, this case report may help future patients and provide a rationale for initiating clinical research in the use of Ra-223 dichloride to treat bone metastasis from breast cancer. A randomized clinical trial is needed to provide evidence of efficacy, safety, and good outcomes.

## Introduction

Hormone-refractory breast cancer metastatic to bone is a clinically challenging disease associated with high morbidity and poor prognosis. Common complications of bone metastasis include skeletal-related events such as pathologic fractures, spinal cord compression, and nerve root compression, as well as hypercalcemia of malignancy. Moreover, bone pain impairs quality of life in patients with bone metastases. Strategies to target bone metastases have included bisphosphonate therapy (zoledronic acid), receptor activator of nuclear factor kappa-B ligand (RANK-L)–directed monoclonal antibody therapy (denosumab), and palliative radiation in addition to systemic therapy [1-4]. A recent major therapeutic advance was the US Food and Drug Administration (FDA) approval of radium-223 (Ra-223) dichloride (Xofigo injection, Bayer HealthCare Pharmaceuticals Inc) alpha particle therapy for the treatment of symptomatic bone metastases in patients with castration-resistant prostate cancer and no known visceral metastatic disease [3,5-8]. Ra-223 is an isotope of radium with an 11.4-day half-life, in contrast with the common isotope Ra-226, discovered by the Curies, which has a 1601-year half-life.

In a preclinical study using a mouse model of breast cancer and bone metastases, Ra-223 dichloride was incorporated into bone matrix and inhibited proliferation of breast cancer cells and differentiation of osteoblasts and osteoclasts (all *P* values < .001) *in vitro*. Ra-223 dichloride also induced double-strand DNA breaks in cancer cells *in vivo*[9]. Moreover, Ra-223 dichloride extended survival duration when administered as a single agent or in combination with zoledronic acid or chemotherapy (doxorubicin) compared with control [9]. On the basis of this strong preclinical rationale, we used Ra-223 dichloride to treat bone metastases in a patient with breast cancer, and the patient derived clinical benefit from the therapy. This case report may help future patients and provide a rationale for initiating clinical research in the use of Ra-223 dichloride to treat bone metastases from breast cancer.

### Clinical case report

A 44-year-old white woman with a history of hypertension, hypercholesterolemia, hypothyroidism, anxiety, and renal stones presented in September 1992 with metastatic breast cancer. At the time of diagnosis, her breast tumor was estrogen receptor–positive, *BRCA1*-negative, and *BRCA2*-negative, and the tumor had spread to 8 of 16 lymph nodes. She received frontline standard chemotherapy with doxorubicin, 5-fluorouracil, methotrexate, and cyclophosphamide for 9 months, followed by oral tamoxifen for 4 years and 6 months.

She was clinically stable until the summer of 2002, when she started to complain of back pain. Magnetic resonance imaging of the spine revealed osseous metastatic disease in the spine involving the T1 vertebral body. She was treated with directed radiation for 5 weeks and then started taking the oral nonsteroidal aromatase inhibitor letrozole, which she continued for 2 years, along with zoledronic acid.

In April 2006, her tumor marker CA27-29 levels increased to 76 U/mL. Magnetic resonance imaging and computed tomography (CT) scans showed progressive disease with multifocal osseous metastatic disease involving the spine. She ceased treatment with oral letrozole and started taking fulvestrant, a selective estrogen receptor downregulator, and she continued taking zoledronic acid. Her CA27-29 levels initially decreased but then increased again to 37 U/mL in November 2006. She continued with the anti-hormonal therapy (fulvestrant and zoledronic acid), adding progesterone cream to her regimen in April and May 2007. In November 2007, her CA27-29 levels decreased to 24 U/mL.

She continued with the same anti-hormonal therapy from 2010 until 2013, when she was once again diagnosed with metastatic disease. In May 2013, she started systemic therapy with the mammalian target of rapamycin (mTOR) inhibitor everolimus in combination with exemestane. She received only 3 cycles, with a starting dose of 2.5 mg orally every day. The 6-week therapy was complicated by shingles and abnormal liver enzymes, and CA27-29 levels markedly increased to 705 U/mL. A CT scan on August 15, 2013, revealed no evidence of visceral metastatic disease but showed stable osseous metastatic disease in the spine. She reported progressive bone pain affecting her back, shoulder, and pelvic regions.

The patient was then referred to The University of Texas MD Anderson Cancer Center Clinical Center for Targeted Therapy to receive molecularly targeted therapy. Pathologic review showed that the spinal tumor cells were strongly positive for estrogen receptor (90%) and moderately positive for progesterone receptor (15%). In addition, *HER-2/neu* protein overexpression was equivocal (score 2+, 80%), and the Ki-67 proliferation index was positive in less than 10% of tumor cells. Consistent with the pathology report, *HER-2/neu* gene amplification according to fluorescence in situ hybridization was reported as equivocal (score 1.83). This immune profile was consistent with a breast tumor origin. A next generation sequencing–based analysis for the detection of frequently reported (hotspot) mutations in a total of 50 genes was performed on DNA extracted from an archival sample from the patient in the MD Anderson CLIA-certified molecular diagnostics laboratory. *PIK3CA* mutation was detected in codon 1047, exon 21 (CAT to CGT) of the *PIK3CA* gene, which would change the encoded amino acid from histidine to arginine (p.His1047Arg). Imaging studies confirmed that the patient had metastatic disease only in her bones, and no visceral disease. Although several trials were available for treatments targeting the *PIK3CA* pathway, she was unfortunately not a candidate for the trials because she had no RECIST measurable disease.

**Figure 1:**
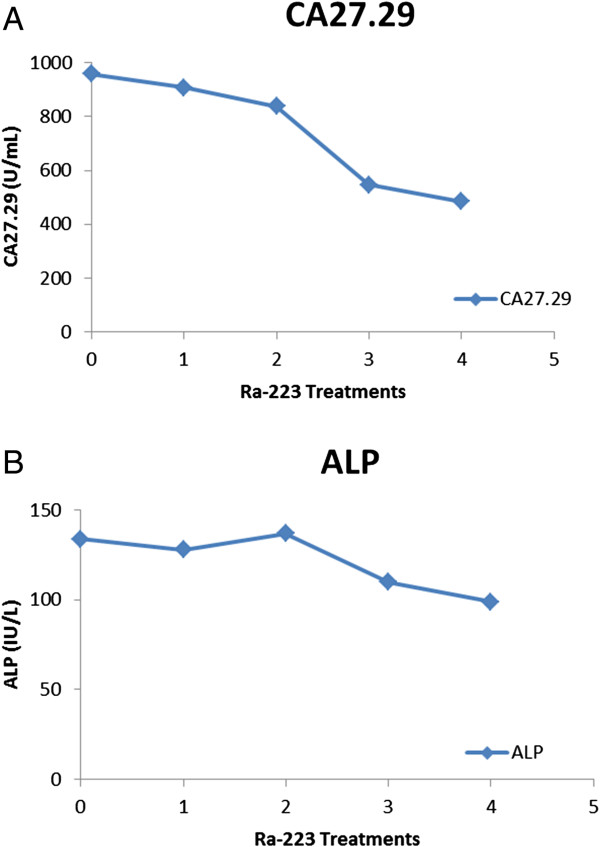
**Graphical representation of tumor marker and alkaline phosphatase over time. A**: Tumor marker CA 27–29 levels after each treatment with radium-223 (Ra-223) dichloride, **B**: Serial alkaline phosphate (ALP) levels after each treatment with Ra-223 dichloride.

She was then referred to Biomedical Research Foundation of Northwest Louisiana for evaluation and treatment with bone-targeted therapy that included Ra-223. Because of the lack of therapy options for hormone-refractory bone metastasis from breast cancer, as well as the strong preclinical data, Ra-223 dichloride therapy was considered. Active osseous metastatic disease without any visceral metastatic disease was confirmed by ^18^F-FDG positron emission tomography (PET)/CT and ^18^F-NaF bone PET/CT imaging prior to Ra-223 consideration. The patient was counseled about the risks and potential benefits of the therapy, the off-label nature of the therapy, and expected adverse events, as well as required precautions to be taken after Ra-223 is administered. Laboratory evaluation was then performed (complete blood count and chemistry profile) to ensure that the patient was eligible for Ra-223 dichloride therapy. After the patient provided informed consent for the off-label use of Ra-223, she was treated with Ra-223 dichloride at a dose of 50 KBq/kg (or 1.35 uCi/kg) every 4 weeks. A total of 6 treatments were planned, and at the time this case report was written, she had completed 4 of the 6 planned treatments. Before each treatment, laboratory evaluation was performed to assess for adverse events related to hematologic parameters and to measure markers (CA27-29 and serum alkaline phosphate). After 2 treatments, ^18^F-FDG PET/CT and ^18^F-NaF bone PET/CT imaging studies were performed to evaluate disease status.The patient reported significant improvement in her pain soon after the first treatment and has reported incremental and sustained improvement in her pain with subsequent treatments. This was accompanied by laboratory evidence of response: CA27-29 levels were 957.6 U/mL at baseline prior to first treatment and have consistently decreased with each treatment, to 483.9 U/mL after 4 treatments. Serum alkaline phosphate levels were 134 IU/L at baseline prior to first treatment and have also consistently decreased with each treatment, to 99 IU/L after 4 treatments. Figure 1 shows a graphical representation of the decreases in CA27-29 (A) and alkaline phosphate levels (B). At the time of this report (after 4 treatments), she reported being pain-free without any analgesics, with a very good quality of life and without any restrictions in her daily activities.

^18^F-FDG PET/CT and ^18^F-NaF bone PET/CT imaging at baseline prior to the first treatment showed multifocal osseous metastatic disease with intense uptake. Repeat qualitative and quantitative ^18^F-FDG PET/CT and ^18^F-NaF bone PET/CT imaging after 2 treatments (but prior to the third treatment) showed an overall mixed change in the lesions, with improvement in some lesions but potential progression in others. The lesions were assessed for their uptake of FDG and NaF (semiquantitatively, using the standardized uptake value [SUV]) as well as for their density on the CT portion of the study (Hounsfield units [HU]).

**Figure 2:**
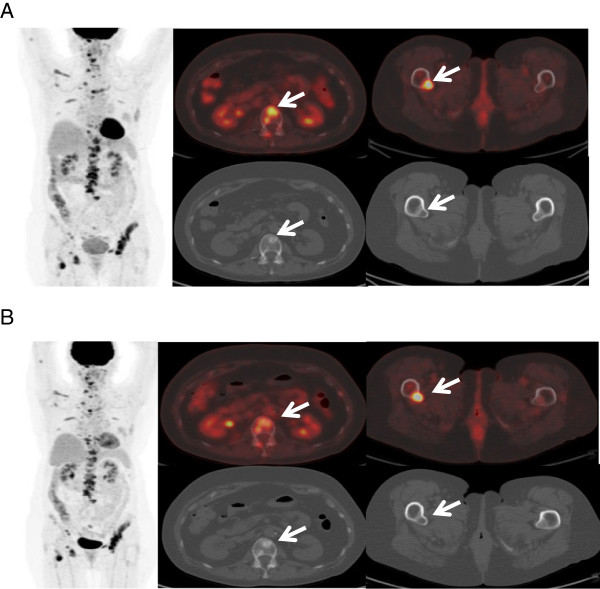
**Imaging study with **^**18**^**F-FDG PET/CT. A**: ^18^F-FDG PET/CT imaging studies at baseline, before administration of radium-223 (Ra-223) dichloride, **B**: ^8^F-FDG PET/CT imaging studies after 2 treatments with Ra-223 dichloride but before the third treatment.

**Figure 3:**
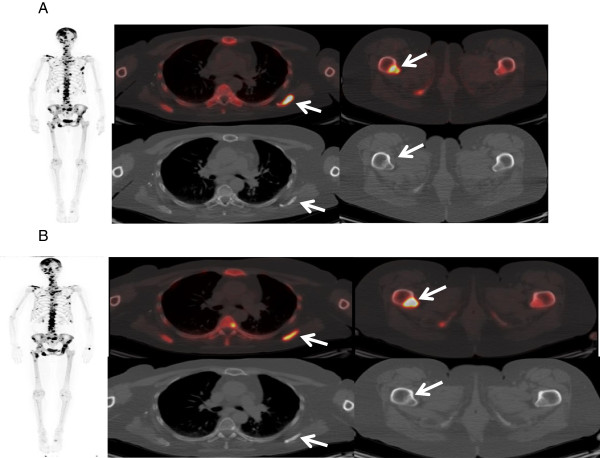
**Imaging study with **^**18**^**Na-F Bone PET/CT. A**: ^18^NA-F bone PET/CT imaging studies at baseline, before administration of radium-223 (Ra-223) dichloride, **B**: ^8^NA-F bone PET/CT imaging studies after 2 treatments with Ra-223 dichloride but before the third treatment.

For the sake of this report, 2 index lesions were selected for analysis on the ^18^F-FDG PET/CT (Figure 2) and ^18^F-NaF bone PET/CT (Figure 3) imaging studies. At baseline, the ^18^F-FDG PET/CT study showed a lesion in the L-2 vertebral body with a maximum SUV of 9.8 and an average SUV of 6.1, and maximum HU of 573 and average HU of 398.9; as well as a lesion in the right proximal femur with a maximum SUV of 12.3 and an average SUV of 7.6, and maximum HU of 348 and average HU of 130.9. On the ^18^F-FDG PET/CT study performed after 2 treatments, the lesion in the L-2 vertebral body appeared less intense and more dense or sclerotic, with a maximum SUV of 7.9 and an average SUV of 4.7, and maximum HU of 636 and average HU of 489.8. This indicates interval improvement. However, the lesion in the right proximal femur appeared larger and more intense, with a maximum SUV of 13.2 and an average SUV of 8.2, and maximum HU of 304 and average HU of 95.1. This indicates potential interval worsening or progression.

Similarly, at baseline, the ^18^F-NaF bone PET/CT study showed a lesion in the left scapula with a maximum SUV of 54.6 and an average SUV of 33.3, and maximum HU of 687 and average HU of 179.6; as well as a lesion in the right proximal femur with a maximum SUV of 31.3 and an average SUV of 17.7, and maximum HU of 431 and average HU of 152.3. On the ^18^F-NaF bone PET/CT study performed after 2 treatments, the lesion in the left scapula appeared less intense and more dense or sclerotic, with a maximum SUV of 21.4 and an average SUV of 13.4, and maximum HU of 757 and average HU of 234.4. This indicates interval improvement. However, the lesion in the right proximal femur appeared larger and more intense, with a maximum SUV of 52.6 and an average SUV of 31.4, and maximum HU of 265 and average HU of 125.7. This indicates potential interval worsening or progression.

## Discussion

To the best of our knowledge, we have reported the first clinical case of hormone-refractory bone metastases from breast cancer responding to alpha-particle Ra-223 therapy outside the context of a clinical trial, soon after US FDA approval of Ra-223 dichloride for the treatment of bone metastases from prostate cancer. After the submission of this case study a phase IIa, nonrandomized study of radium-223 dichloride in advanced breast cancer patients with bone-dominant disease was also published [10]. Twenty-three advanced breast cancer patients with relapsed breast cancer in the bone were administered radium-223 (50 kBq/kg IV) every 4 weeks for 4 cycles. It was demonstrated that Radium-223 was safe in breast cancer in addition to clinically target areas of increased bone metabolism [10].

Cancer-related mortality is often the result of metastatic disease, especially metastases in the lungs, brain, liver, and bone. For bone metastases, bisphosphonates and RANK-L monoclonal antibody are the mainstay targeted therapies, in addition to the systemic therapy armamentarium [4]. Radiotherapy is palliative, and previous beta particle therapies such as samarium are toxic to bone marrow and have limited effectiveness because they cannot be combined with other systemic therapies.

Ongoing clinical trials of treatments involving radium include expanded use of cabazitaxel for the treatment of prostate cancer, Alpharadin for the treatment of bone-dominant metastatic breast cancer no longer considered suitable for hormone therapy (NCT01070485), and a phase I dose escalation trial of monthly intravenous Ra-223 dichloride for the treatment of osteosarcoma (NCT01833520).

For the patient in the current case report, we observed a positive treatment effect of Radium 223 in hormone-refractory bone metastases from breast cancer. Close follow-up with imaging and monitoring of serial tumor markers is planned. Our initial assessment indicated a very good clinical response, with a significant drop in tumor and alkaline phosphatase marker levels. Imaging results were more mixed, and further investigation is needed to determine the potential for the flare phenomenon and the most optimal imaging modality.

## Conclusion

We have shown that Ra-223 dichloride can be administered without toxicity in a patient with hormone-refractory bone metastasis from breast cancer at the US FDA–approved dose for prostate cancer. Furthermore, because the treatment did not cause any drop in hematologic parameters, it has the potential to be combined with other radiosensitizing therapies, which may include chemotherapy or targeted therapies. Given that Ra-223 dichloride is already available on the market, this case report may help future patients and provide a rationale for initiating clinical research in the use of Ra-223 dichloride to treat bone metastasis from breast cancer. A randomized clinical trial is needed to provide evidence of efficacy, safety, and good outcomes.

• Hormone-refractory bone metastasis from breast cancer is associated with high morbidity, poor prognosis, and impaired quality of life owing to pain and skeletal-related events.

• The US Food and Drug Administration recently approved radium-223 (Ra-223) dichloride (Xofigo injection, Bayer HealthCare Pharmaceuticals Inc) alpha particle therapy for the treatment of symptomatic bone metastases in patients with castration-resistant prostate cancer.

• We used Ra-223 dichloride to successfully treat hormone-refractory bone metastases in a patient with breast cancer.

• Our initial assessment indicated a very good clinical response, with a significant drop in tumor and bone turnover marker levels; however, imaging results were more mixed, and further investigation is needed.

• Ra-223 dichloride can be safely administered in a patient with hormone-refractory bone metastases from breast cancer.

• A randomized clinical trial is needed to provide evidence of efficacy, safety, and good outcomes.

### Patient disclosure

The authors state that they have obtained informed consent from the patient for the administration of radium off label.

## Competing interests

Amol Takalkar, MD, is on the Bayer Healthcare Speaker Bureau and has served on the Advisory Committee for Bayer Healthcare Pharmaceuticals. Vivek Subbiah, MD has research support from Bayer and served on medical advisory board. All other authors have no relevant affiliations or financial involvement with any organization or entity with a financial interest in or financial conflict with the subject matter or materials discussed in the manuscript. This includes employment, consultancies, honoraria, stock ownership or options, expert testimony, grants or patents received or pending, or royalties. No writing assistance was used in the production of this manuscript.

## Authors’ contributions

All authors contributed to writing the manuscript. AT, VS conceived the manuscript. AT, SA, VS analysed the data. VS, and AT wrote the paper. VS provided targeted therapy expertise and AT provided nuclear medicine expertise. All authors read and approved the final manuscript.
